# Retraction: PKM2 overexpression protects against 6-hydroxydopamine-induced cell injury in the PC12 cell model of Parkinson’s disease *via* regulation of the brahma-related gene 1/STAT3 pathway

**DOI:** 10.1039/d1ra90036f

**Published:** 2021-01-26

**Authors:** Laura Fisher

**Affiliations:** Royal Society of Chemistry Thomas Graham House, Science Park, Milton Road Cambridge CB4 0WF UK advances-rsc@rsc.org

## Abstract

Retraction of ‘PKM2 overexpression protects against 6-hydroxydopamine-induced cell injury in the PC12 cell model of Parkinson’s disease *via* regulation of the brahma-related gene 1/STAT3 pathway’ by Lei Jiang *et al.*, *RSC Adv.*, 2019, **9**, 14834–14840, DOI: 10.1039/C9RA01760G.

The Royal Society of Chemistry hereby wholly retracts this *RSC Advances* article due to concerns with the reliability of the data. The images in the article, and the raw data provided by the authors, were screened by an image integrity expert.

The published western blots in Fig 1B showed signs of manipulation, and the raw data provided by the authors for Fig 1B also showed evidence of extensive manipulation. In addition, the raw data provided for Fig 4 was not genuine as it consisted of the bands being placed onto false backgrounds. Therefore, the raw data provided by the authors cannot be used to validate the published data.

Given the significance of the concerns about the validity of both the data in the article and the raw data provided by the authors, the findings presented in this paper are not reliable.

The authors have been informed but have not responded to any correspondence regarding the retraction.

Signed: Laura Fisher, Executive Editor, *RSC Advances*

Date: 15^th^ January 2021

## Supplementary Material

